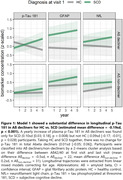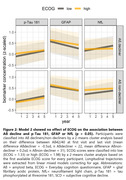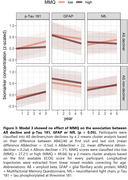# Reduced plasma Aß42/40 in individuals with SCD predicts increasing plasma p‐Tau 181

**DOI:** 10.1002/alz.090959

**Published:** 2025-01-09

**Authors:** Laura Göschel, Peter Koertvelyessy, Patty Hoede, Charlotte Teunissen, Agnes Flöel

**Affiliations:** ^1^ Charité – Universitätsmedizin Berlin, corporate member of Freie Universität Berlin and Humboldt‐Universität zu Berlin, Department of Neurology, Charitéplatz 1, Berlin Germany; ^2^ Charité – Universitätsmedizin Berlin, corporate member of Freie Universität Berlin and Humboldt‐Universität zu Berlin, NeuroScience Clinical Research Center, Charitéplatz 1, Berlin, Berlin Germany; ^3^ Charité – Universitätsmedizin Berlin, corporate member of Freie Universität Berlin and Humboldt‐Universität zu Berlin, Department of Neurology, Charitéplatz 1, Berlin, Berlin Germany; ^4^ German Center for Neurodegenerative Diseases (DZNE), Magdeburg Germany; ^5^ Neurochemistry Laboratory, Department of Laboratory Medicine, Vrije Universiteit Amsterdam, Amsterdam UMC, Amsterdam, Netherlands, Amsterdam Netherlands; ^6^ Neurochemistry Laboratory, Department of Laboratory Medicine, Vrije Universiteit Amsterdam, Amsterdam UMC, Amsterdam, North Holland Netherlands; ^7^ Department of Neurology, University Medicine Greifswald, Greifswald Germany; ^8^ German Centre for Neurodegenerative Diseases (DZNE), Standort Rostock/Greifswald, Greifswald Germany

## Abstract

**Background:**

In Alzheimer’s disease (AD), the decline of plasma Aß42/40 occurs before cognitive decline, presenting a potential early screening tool. However, the factors leading to the progression of the disease, specifically the increase in plasma p‐Tau 181, glial fibrillary acidic protein (GFAP), and neurofilament light chain (NfL), remain unclear. This study investigates whether perceived cognitive impairment is associated with downstream biomarker changes in individuals with decreasing Aß42/40.

**Method:**

Plasma was longitudinally collected from a study population of cognitively healthy controls (HC, n=21) and individuals with subjective cognitive decline (SCD, n=32) for up to five time points over up to five years. Plasma biomarkers were measured using Simoa HD‐X (Billerica, USA) with the commercially available kits NEUROLOGY 4‐PLEX E and p‐Tau 181 advantage kit V2. A 2‐means cluster analysis classified the participants as Aß decliners/non‐decliners based on their plasma Aß42/40 change (difference between plasma Aß42/40 at the first visit and last visit). The self‐reported Everyday Cognition (ECOG) and Multifactorial Memory Questionnaire (MMQ) provided two continuous measures of subjective cognition. Using linear mixed models, we examined z‐scaled longitudinal plasma p‐Tau 181, GFAP and NfL for Aß decliners and non‐decliners with a diagnosis of SCD (model 1), elevated scores in ECOG (model 2) or decreased scores in MMQ (model 3).

**Result:**

The cluster analysis separated the participants into Aß decliners (plasma Aß42/40 change=‐0.5sd, n=22) and non‐decliners (Aß change=0.2sd, n=31). Model 1 showed a substantial difference in longitudinal p‐Tau 181 in Aß decliners for HC vs. SCD (estimated mean difference=‐0.19sd, p<0.001). A yearly increase of plasma p‐Tau 181 in Aß decliners was found only for SCD (0.10sd [0.03; 0.18], p=0.008) but not HC (‐0.09sd [‐0.17; ‐0.01], p=0.028) participants. Neither model 2 nor model 3 showed relevant effects of ECOG or MMQ on longitudinal plasma p‐Tau 181, GFAP or NfL, in neither Aß decliners nor non‐decliners.

**Conclusion:**

Our findings suggest a predictive value of the diagnosis SCD for pathologic progression in participants with declining plasma Aß42/40. Our study contributes to a better understanding of early pathological changes of AD and highlights the use for a multi‐modal diagnostic approach involving both objective biomarkers and professional evaluation.